# Racialized Heteroscedasticity in Neuroimaging Features, Behavior Measures, and Neuroimaging-Based Predictive Models

**DOI:** 10.21203/rs.3.rs-9557211/v1

**Published:** 2026-06-28

**Authors:** Christopher Fields, Matthew Rosenblatt, Joseph Aina, Jannat Thind, Annie Harper, Chyrell Bellamy, Xin Zhou, Alexandra Potter, Hugh Garavan, Nicholas Allgaier, Micah Johnson, Raimundo Rodriguez, Fahmi Khalifa, Deanna Barch, Dustin Scheinost

**Affiliations:** Yale School of Medicine; Yale University; Morgan State University; Yale School of Medicine; Yale School of Medicine; Yale School of Medicine; Yale School of Public Health; University of Vermont; Departments of Psychiatry and Psychology, University of Vermont, 05405 Burlington, Vermont, USA; University of California-Los Angeles Health; Yale University; Morgan State University; Washington University in St. Louis; Yale School of Medicine

**Keywords:** Resting-state fMRI, racialized heteroscedasticity, connectome-based predictive modeling, neuroimaging, predictive modeling, behavioral measures

## Abstract

Neuroimaging studies rarely test whether the variance structure is equivalent across population subgroups. Here, in 4,736 participants from the Adolescent Brain Cognitive Development (ABCD) cohort, we examine racialized heteroscedasticity (i.e., differences in variance across racialized groups) in neuroimaging and behavioral data and test how these differences in variance propagate into predictive modeling. Across neuroimaging modalities, behaviors, and predictive frameworks, variance differences exhibited consistent patterns, indicating that variance structure is a stable property across domains within the dataset. Simulation analyses demonstrated that such differences directly induce subgroup disparities in prediction error and reliability, even in the absence of mean differences. Across neuroimaging modalities, multiple measures demonstrated greater variance in Black participants, particularly in functional imaging modalities. Similar variance patterns were observed in behavioral measures, and predictive models exhibited greater residual dispersion and prediction variance in Black participants even when overall performance metrics were comparable. These findings position variance structure, rather than central tendency, as a critical determinant of model performance, generalizability, and reliability across diverse populations.

## Background

Recent studies have highlighted the importance of considering racial and ethnic differences in neuroimaging research.^1,2^ Differences in neuroimaging and behavior may reflect unequal exposure to stress, environmental adversity, and access to supportive resources across development.^3–17^ These influences are not uniformly experienced within racial groups, which may result in greater variability in neural and behavioral phenotypes.^18–23^ Rather than attributing such differences to intrinsic neurobiology, contemporary frameworks emphasize racialization as a social process shaping lived experience and exposure across development.^3–6,24^

Heteroscedasticity is a well-established statistical concept but has received limited attention in neuroimaging studies.^25,26^ This statistical phenomenon manifests when the variability in predictors or outcomes differs systematically between groups.^26^ In predictive modeling, the assumption of homoscedasticity (equal variance) is often made, which may lead to biased or less accurate predictions when it is violated.^27,28^ For example, brain-behavior models trained on data exhibiting heteroscedasticity may be disproportionately shaped by lower-variance groups. In turn, generalizability may be limited, obscuring meaningful variability in higher-variance populations.^29,30^ This can also lead to unreliable risk assessments and inappropriate treatment recommendations in clinical settings.^31,32^ In line with other clinical models (e.g., models for kidney health, cardiovascular health, maternal risk, etc.),^31,33–40^ neuroimaging models––including models trained solely on Black American data––perform worse for Black versus White participants.^41,42^ Given the growing application of neuroimaging-based statistical and predictive models, it is essential to determine whether variance structures differ across racialized groups. Violations of homoscedasticity may alter both inference validity and model reliability.

This study addresses a critical yet largely untested assumption that the variances of brain and behavioral variables are the same across subgroups. We operationalize potential variance differences between Black and White participants as “racialized heteroscedasticity” to emphasize that these differences likely reflect how socially structured exposures may lead to nonconstant variance in large datasets. Using the Adolescent Brain Cognitive Development (ABCD) Study, we examine racialized heteroscedasticity in neuroimaging and behavioral measures and test whether these differences in variance propagate into brain-behavior prediction. ABCD provides an unparalleled opportunity to examine this question because its scale allows for the evaluation of variance structure, not only means, across multiple modalities and behaviors. By focusing on variance differences between Black and White participants, we seek to shed light on potential disparities in prediction certainty. Understanding these differences is crucial not only for building more equitable predictive models but also for interpreting large-scale neuroimaging results when variance is different across groups.

## Results

### Overview

In this study, we test whether a core assumption underlying population neuroimaging, that variance structure is comparable across racialized groups, is violated across brain, behavior, and predictive modeling. We proceed in four stages. First, we quantify variance differences between Black and White participants across six neuroimaging modalities in the Adolescent Brain Cognitive Development (ABCD) dataset: diffusion-weighted imaging (DWI), T1-weighted structural MRI (SMRI), resting-state fMRI (rsfMRI), and task-based fMRI from the Monetary Incentive Delay (MID), Stop-Signal Task (SST), and EN-Back paradigms. Second, we assess variance differences across a broad battery of behavioral outcomes. Third, we evaluate whether and how these variance patterns manifest in machine learning predictions, focusing on connectome-based predictive modeling (CPM). Complementary models (kernel ridge regression, random forest, and artificial neural network) are shown in the Supplement. Fourth, we use simulations to demonstrate how group differences in predictor and outcome variance affect prediction accuracy in linear regression models. After quality control and data filtering, our final sample included 4,736 participants (3,892 White and 844 Black) with complete imaging and demographic data **(Table S1).** Across these analyses, we focus on whether variance structure itself constitutes a meaningful and previously underappreciated source of divergence in brain-behavior inference.

Although these analyses are conducted within a single cohort, they span multiple independent axes, including neuroimaging modalities (structural and functional), behavioral phenotypes across cognitive and affective domains, and predictive modeling frameworks. These domains differ substantially in measurement, dimensionality, and statistical structure. Thus, convergence of variance patterns across these axes provides internal evidence that variance structure is not specific to a single data type or analytical approach but instead reflects a broader and reproducible property of the dataset.

### Racial Differences in Variance for Neuroimaging Features

For the neuroimaging results, we first present uncorrected findings to characterize the raw variance structure propagated by typical multivariate pipelines. We then evaluate robustness using false discovery rate (FDR) correction and accounting for covariates (age, self-reported sex, family income, head motion), site differences, and imbalanced sample sizes by race.

Across all modalities, 12.44–46.36% of neuroimaging features (e.g., Fractional Anisotropy (FA) in the white matter or an edge in a connectome) exhibited significant differences (p < 0.05, uncorrected) in variance between Black and White participants (Table 1, **Figure S1, Figure S2**). Among resting-state and task-based fMRI, more features showed significantly greater variance in Black than in White participants (62.37–91.69%, *χ*^**2**^s > 350, ps < 0.001). In contrast, DWI exhibited a significant number of features with greater variance in White versus Black participants (78.57%, *χ*^**2**^ = 22.86, p < 0.001). For SMRI, there were no differences in the number of higher variance features between Black and White participants (*χ*^**2**^ = 0.02, p = 0.89). This pattern provides a complementary contrast to the other modalities, where variance is greater in Black participants, and supports the interpretation that prediction behavior follows variance structure rather than group identity. Consistent with this, simulations show that increasing variance in any group produces the same directional changes in prediction error and variability. Importantly, this cross-modality consistency suggests that observed variance differences are not specific to a single imaging representation but emerge across distinct measurement frameworks.

Although applying all conservative corrections simultaneously (covariate regression, site correction, balanced sample sizes, and FDR correction) greatly reduced the number of significantly different features across modalities, the remaining significant features all exhibited higher variance in Black participants (Table 1). We additionally examined each correction strategy individually and observed broadly similar overall trends (Table 2). Across correction strategies, fMRI modalities consistently demonstrated higher variance in Black participants. DWI and SMRI results were less consistent across correction approaches. In DWI, significant variance differences persisted across corrections, although whether variance was greater in Black or White participants depended on the specific correction strategy applied. SMRI often exhibited few or no significant variance differences. Thus, while conservative correction strategies reduced the overall number of significant findings, they did not eliminate variance structure differences across groups, indicating that standard correction approaches alone may be insufficient to fully account for subgroup differences in variance. Moreover, variance differences that do not survive stringent correction thresholds may still meaningfully influence multivariate and machine learning analyses, which aggregate many small effects.

### Neuroanatomical Distribution of Variance Differences

**Figure 1:**
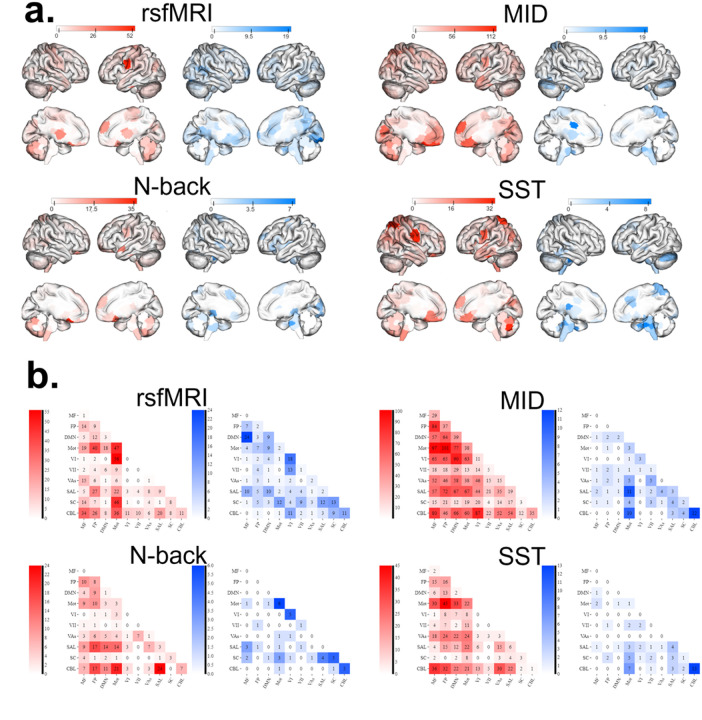
Distribution of Levene’s test significant differences across the brain. Levene’s test was performed to compare significantly different edges between Black and White participants in resting-state fMRI data. The edges with significantly greater variance (no correction) in Black compared to White participants were summarized across brain regions (**A**) and brain networks (**B**) in red. Similarly, the edges with significantly greater variance in white compared to Black participants were summarized across brain regions (**A**) and brain networks^75,76^ **(B**) in blue. MF, medial-frontal; FP, fronto-parietal; DMN, default mode; MOT, motor; VI, visual I; VII, visual II; VAs, visual association; SAL, salience; SC, subcortical; and CBL, cerebellum.

Given the robustness of fMRI results across various corrections, we focused on characterizing the spatial distribution of significantly different functional connectivity edges. For models without any corrections, regions in the cerebellar network commonly exhibited higher variance in Black participants across all modalities (Fig. 1). Motor regions showed greater variance in Black participants during resting state, MID, and SST, but not during the EN-back task. Consensus regions were defined as regions with at least 10 edges showing higher variance in Black than in White participants in resting-state and task-based fMRI **(Figure S3A)** and were distributed across the salience and frontoparietal networks **(Figure S3B)**.

### Racial Differences in Variance for Behavioral Measures

In addition to neuroimaging, we analyzed variance differences across 36 behavioral measures from the ABCD dataset, using Levene’s test to assess differences in variance between Black and White participants. These measures spanned cognition, personality traits, emotional regulation, and behavioral health. Like the imaging analyses, covaried models were tested, controlling for age, self-reported sex, and family income. To account for multiple comparisons, FDR correction was applied to the results. Of the 36 behavioral measures analyzed, 30 measures exhibited significant differences in racial variance after FDR correction (33 uncorrected; Table 3). Of these, 27 showed greater variance in Black participants compared to White participants. Among these measures, several from the Child Behavior Checklist (e.g., rule-breaking, aggression, and attention problems) and personality domains (e.g., sensation-seeking and impulsivity) exhibited the most pronounced differences. Conversely, three measures exhibited greater variance in White participants. These findings suggest racialized heteroscedasticity exists across multiple psychological and cognitive domains, even after accounting for demographic covariates and rigorous false positive control. The consistency of these variance differences across a diverse set of 36 behavioral measures further supports the interpretation that variance structure is not confined to a narrow set of phenotypes, but extends across cognitive, emotional, and behavioral domains.

### Racial Differences in Neuroimaging-based Predictive Models

Because both neuroimaging features and behavioral outcomes exhibited variance differences, we next asked whether these differences propagated into the stability of brain-behavior predictions. Using Connectome-based Predictive Modeling (CPM) and five-fold cross-validation with 10% feature selection, we fit models to predict the 36 behavioral measures from whole-brain resting-state functional connectivity. Among these 36 measures, 20 had correlations between observed and predicted behaviors that were at least modest (*r* > 0.1) and significant (p < 0.05) based on permutation testing (Table 4). We also report the prediction performance stratified by race (**Table S2**). Generally, results were conflicting with both correlation and mean square error (MSE) being higher in Black participants. Of these 20 phenotypes, 15 (75%) had greater correlation between observed and predicted, and 18 (90%) had greater MSE for Black participants. The difference in correlation and MSE between Black and White participants was significantly negatively correlated (ρ=−0.49, p = 0.03), indicating that phenotypic predictions showing better correlations between observed and predicted values for Black participants were the same predictions showing worse MSE for Black participants. While higher correlations suggest improved fit, higher MSE indicates greater prediction error, highlighting conflicting signals across performance metrics. Thus, single measures of prediction accuracy alone can obscure subgroup differences in prediction quality.

**Figure 2:**
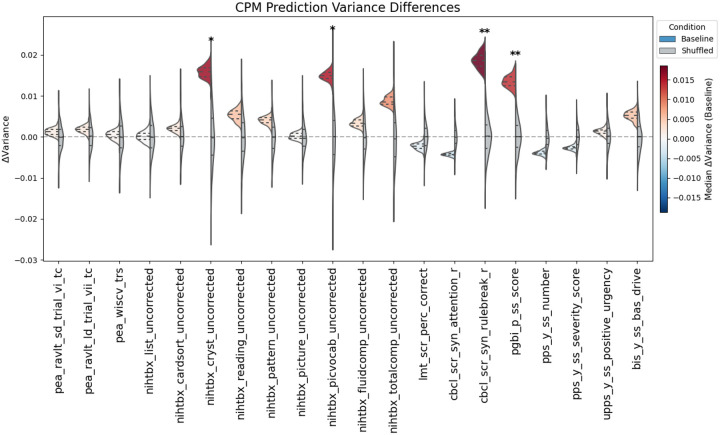
Variances differences in predicted values by race. The figure presents results for predictive models with at least modest (*r*>0.1) and significant (p<0.05) predictions. In the left violin plot, the observed variance difference (variance in predicted values of Black participants minus variance in predicted values of White participants) is shown, colored by median variance difference. In the right violin plot, the null distribution of variance differences resulting from 1000 permutations shuffling the race is shown. *p<0.05. **FDR-significant.

To better explain these group differences in standard measures of prediction accuracy, we next investigating how the underlying the variance of predicted values race difference by race. Applying a permutation-based scheme to assess these differences (Fig. 2), we identified FDR-corrected (p < = 0.003) significant differences in variance in two of the 20 models (cbcl_scr_syn_rulebreak_r, pgbi_p_ss_score), both of which exhibited higher variance in Black participants. Nominally significant (p < 0.05) variance differences in prediction values were present in models from four phenotypes, all of which had higher variance in Black participants. Most differences in prediction variance were positive (16/20) (Fig. 2), indicating a greater spread of predicted values for Black participants. Using a binomial test, this difference in the number of positively and negatively skewed variance differences was significantly higher than what would be expected by chance (p = 0.012). We ran several sensitivity analyses to assess the robustness of these findings as well as formal tests of heteroscedasticity in brain-behavior relationships are shown in the Supplement. Together, these analyses indicate that greater prediction variances in Black participants are robust to analytical choices and are consistently aligned with upstream variance differences in neuroimaging features and behavioral measures.

### Mechanistic Basis: How Variance Structure Alters Prediction Behavior

To provide a mechanistic account of the empirical patterns observed above, we simulated a simplified regression scenario, mirroring the variance differences observed in the ABCD data, but without any confounding variables. Specifically, we asked whether greater differences in variance in either predictors (X) or outcomes (Y) could systematically degrade prediction accuracy when groups are pooled in a single model. In the simulation, two groups with the same relationship between X and Y were pooled together, with the variance of X and Y in group 2 allowed to be greater than those variances in group 1. As shown in **Figure S5**, a greater Y variance in group 2 led to increased MSE compared to group 1. Given that variance and MSE are both measures of spread from a data point, it is intuitive that data with a greater spread (i.e., variance) will lead to greater spread in predictions (i.e., MSE). However, this result highlights that structural differences in variance for subgroups will lead to one subgroup exhibiting a higher MSE and the appearance of a worse performing model. Greater X variance in group 2 led to increased correlation between observed and predicted compared to group 1, whereas greater Y variance decreases this correlation. Notably, when increased X and Y variances for group 2 were similar, no changes in correlations compared to group 1 were observed. Further, X variance seemed to have smaller impact on MSE than correlation. These results provide a mechanistic explanation for the empirical findings observed across neuroimaging, behavioral, and predictive analyses, demonstrating how variance differences alone can generate subgroup disparities in prediction performance. They show how differences in variance structure can lead to conflicting model performance (increased MSE and correlation between observed and predicted values) for groups with higher variance. They also show how certain variance structures can cancel each other, masking potential biased model performance. Overall, differences in variance structure directly affect standard measures of prediction accuracy and, likely, changes how carefully model success should be interpreted.

## Discussion

Our study reveals widespread variance differences across neuroimaging features, behavioral measures, and predictive-model outputs in the ABCD Study. Importantly, the consistency of these differences across multiple domains indicate that variance structure is a stable property across distinct analytical context. These findings have important implications for neuroimaging research, particularly in the context of diverse populations and the development of predictive models of brain-behavior associations.^32,43,44^ More broadly, these findings expose an incorrect, but largely untested assumption in population neuroimaging: that variance structure is sufficiently similar across subgroups for standard inferential and predictive workflows to be interpreted in the usual way. These findings are derived from a single cohort, and we do not claim that the magnitude or direction of variance differences will generalize identically across all populations or developmental stages. Rather, our results demonstrate that variance structure is a critical and underexamined dimension that can systematically differ across subgroups and directly influence predictive modeling behavior. Our results suggest that heteroscedasticity is not only an equity concern but also a source of instability in neuroimaging-based prediction. A consistent pattern of higher predictive variance for Black participants emerged across multiple modeling approaches (CPM, kernel ridge regression, random forest, and artificial neural network) and behavioral measures. These results suggest that the model outputs are less reliable and less stable, potentially limiting their utility across diverse populations. In addition, these variance differences lead to conflicting changes in two popular measures of prediction accuracy (e.g., correlations and MSE), potentially masking meaningful subgroup differences in prediction quality. Increased uncertainty has implications for potential clinical translation and precision medicine. If not properly accounted for, it could exacerbate known health disparities by using unreliable and biased predictive models.^45,46^ Similar differences in variances between other minoritized groups (e.g., rural vs urban, high vs low SES) likely exist and should be investigated to fully characterize potential sources of inequities. Addressing these challenges will require improving data representation and improved methodological considerations, such as integrating fairness constraints into model training and exploring algorithms that better capture the variability of underrepresented groups.

Several non-mutually exclusive mechanisms may contribute to the observed variance differences. First, differential exposure to environmental and social stressors may increase heterogeneity within subgroups. Second, higher-dimensional data representations (e.g., functional connectivity) may amplify small differences in variability. Statistical aggregation across groups with differing variance structures may introduce instability in model estimates. While these mechanisms are not directly tested here, they provide a framework for interpreting variance differences as a property of structured exposure rather than intrinsic group characteristics. Our results suggest a mechanism to explain bias in predictive models, grounded in variance structure rather than differences in central tendency. Several works have highlighted that neuroimaging-based predictive models perform worse in minoritized populations^41,42^. This worse performance is often attributed to lower representation in training data^47^. In other words, the minoritized populations are underrepresented in the training data, and the model cannot learn characteristics specific to that population. However, balancing training data does not completely mitigate these differences. Our results highlight that accounting for variance differences in features or outcomes might be a path forward to reducing model bias. Notably, many of the observed group differences in prediction uncertainty arise despite modest or inconsistent mean differences, underscoring that variance structure, rather than central tendency, may be the more consequential property for model reliability. These findings, together with the simulation results, indicate that variance asymmetry alone is sufficient to produce divergent model behavior across subgroups, even when underlying relationships between predictors and outcomes are identical. Researchers must be cautious in interpreting and communicating findings related to racialized heteroscedasticity to avoid reinforcing harmful stereotypes or biological determinism.^48^ It is crucial to emphasize that these differences likely reflect a complex interplay of social, environmental, and biological factors rather than inherent racial characteristics. Responsible reporting of these results should always contextualize them within the broader societal and historical factors that contribute to health disparities.^49^ We use the term “racialized heteroscedasticity” to emphasize that observed variance differences are not inherent to race itself, but reflect how socially structured exposures shape variability in large-scale developmental data. For example, a smaller proportion of measures had greater variance in White participants, indicating that racialized experiences may influence a broad range of participants. Overall, it remains important to consider variability patterns across racialized groups for specific constructs.

In addition to machine learning and predictive modeling, heteroscedasticity is an issue for conventional statistical inference. A conservative approach that applied covariate regression, site correction, and balanced sample sizes across racial groups reduced racialized heteroscedasticity in neuroimaging features. These results suggest that rigorous corrections, which are already common when using ABCD and other large-scale datasets, could be a first-line approach to mitigate these differences. However, variance differences persisted and were common across behavioral measures, even with these corrections. Further, some of the correction approaches are impractical. For example, balancing analyses across racial groups is unlikely to reflect the local demographics of a study or the US population. Additionally, we only considered two racial groups. Balancing all racial groups is even more impractical. Overall, it remains unclear how heteroscedasticity affects existing research. Together, these findings suggest that variance structure should be regularly examined across subgroups (such as race), rather than treated as a secondary assumption considered only after the main results are reported.

While the exact source of racialized heteroscedasticity remains unknown, our results revealed several patterns across modalities. Racialized heteroscedasticity was more pervasive in functional modalities (resting-state and task-based fMRI) than structural modalities (Table 2). The greater variability in connectivity patterns among Black participants persisted even after our correction strategies. In contrast, structural modalities (DWI and SMRI) exhibited more modest and less consistent differences across correction strategies. These results may reflect that functional connectivity is more affected by race-related factors, which could include experiences of racial discrimination, differences in environmental exposures, or other sociocultural influences.^4,50–53^ Alternatively, they may be a function of the type of data selected. For example, the functional data were more highly dimensional than structural data (35,778 vs 151). Subtle differences in variance patterns may be averaged out at lower spatial scales, leading to less consistent results across correction strategies. High-dimensional structural connectivity, rather than regional FA, may show more consistent patterns of heteroscedasticity. Future work should continue to investigate how heteroscedasticity arises across the numerous representations of neuroimaging data.

While our study provides important insights into racialized heteroscedasticity for neuroimaging studies, several limitations should be considered. First, the binary categorization of race (Black vs. White) does not capture the full spectrum of racial and ethnic diversity. Future studies should examine these patterns across more diverse racial and ethnic groups. Second, while we controlled for several demographic and socioeconomic factors, there may be other unmeasured confounding variables that contribute to the observed differences in variance. More comprehensive assessments of the exposome and cultural factors, particularly those gathered through community-engaged research, could provide additional insights.^54–56^ Similarly, after strict data quality controls, there was a loss of data across between groups, with a greater percent of Black participant removed for lower data quality. This is common, and newer approaches are needed to maintain strict quality control with better sample retention. Third, we examined several popular neuroimaging data types. However, many features can be extracted from these data (e.g., task activations, surface area). Similarly, several other neuroimaging modalities, such as magnetic resonance spectroscopy (MRS), Positron Emission Tomography (PET), or Functional near-infrared spectroscopy (fNIRS), exist. Further investigations using other features and modalities are needed. Fourth, greater variance of predicted values in Black relative to White participants could reflect greater variance in imaging or behavioral data, or both. Future work should focus on disentangling the specific mechanism by which predictive models lead to variance differences.

In conclusion, our study highlights racialized heteroscedasticity in neuroimaging and behavioral data that propagates into brain-behavior associations. These findings underscore the importance of considering potential variance structure in population neuroimaging and, more generally, predictive modeling. Heteroscedasticity in neuroimaging features and behavioral outcomes also provide a mechanism as to why neuroimaging-based predictive models can be biased against minorized populations, even when training data are balanced. Other areas of machine learning may similarly benefit from focusing on variance structure, rather than central tendency, to improve model reliability. By acknowledging and addressing these differences, more equitable and accurate predictive models can be developed. By doing so, we can advance our understanding of brain-behavior associations across diverse populations in clinical and research settings.

## Methods

### Study Population

Data for this study were obtained from the Adolescent Brain and Cognitive Development (ABCD) Study, annual release 5.1 (DOI: 10.15154/z563-zd24). The ABCD Study is a large-scale, longitudinal initiative that recruited 11,878 children aged 9–10 from 22 sites across the United States. Using the baseline data, our analysis focused on participants who self-identified as either White (n = 3,892) or Black (n = 844) to allow for robust statistical comparisons **(Table S1)**. Inclusion required complete baseline data for all relevant measures, including demographics (age, gender, parental income), neuroimaging, and the absence of severe motion artifacts. The distribution of the final sample across income levels and data collection sites is illustrated in **Figure S5**. For analyses involving only behavioral data, we included more participants (White: n = 6,913, Black: n = 2,094) than the imaging-specific analyses.

### Neuroimaging Data Processing

rsfMRI data were processed using BioImage Suite (v.3.01),^57^ as described in our previous work^57^ (and duplicated as follows). This preprocessing included regression of covariates of no interest from the functional data, including linear and quadratic drifts, mean cerebrospinal fluid signal, mean white matter signal and mean global signal. Additional motion control was applied by regressing a 24-parameter motion model, which included six rigid body motion parameters, six temporal derivatives and the square of these terms, from the data. Subsequently, we applied temporal smoothing with a Gaussian filter (approximate cut-off frequency = 0.12 Hz) and grey matter masking, as defined in common space.^57^ Then, the Shen 268-node atlas^57^ was applied to parcellate the denoised data into 268 nodes. Finally, we generated functional connectivity matrices by correlating each time series from pairs of nodes and applying the Fisher transform. In cases where multiple functional connectomes were available, the run with the lowest motion was used. Data were excluded for poor data quality (for example, artefacts in T1-weighted images, misaligned registrations), missing nodes due to lack of full brain coverage, high motion (> 0.2 mm mean frame-wise displacement) or missing phenotypic data.

Task-based fMRI data (MID, SST, and EN-Back) were processed using the same preprocessing pipeline as rsfMRI. For task data, denoised BOLD time series were extracted from each task run after standard preprocessing, without fitting task-specific general linear models (GLMs). Node-wise time series were averaged across the full task session and used to generate task-based functional connectivity matrices in an identical manner to resting-state data. This approach was chosen to ensure consistency across modalities and to allow direct comparison of variance structure in functional connectivity independent of task-evoked mean activation differences.

The ABCD release 5.1 provides preprocessed data for DWI and SMRI. Details of the processing methods are previously described.^58^ For DWI, we downloaded data representing average FA within white matter for the 148-region Destrieux atlas (ABCD table name: “mri_y_dti_fa_fs_wm_dst”).^59^ For SMRI, data was downloaded for the same atlas (ABCD table name: “mri_y_smr_vol_dst”). We investigated each of the 148 regions, as well as mean data values in the left hemisphere, right hemisphere, and whole brain. For both DWI and SMRI analyses, participants were excluded based on ABCD’s recommended inclusion criteria, as previously described.^58^

### Racial Differences in Variance for Neuroimaging Features

To test for differences in variance between Black and White participants, Levene’s test for the equality of variances was conducted across multiple neuroimaging and behavioral modalities in MATLAB (https://www.mathworks.com/help/stats/vartestn.html), using absolute deviations of the data from their group means.^27,60^ Specifically, Levene’s test was applied to each of the 35,778 unique connectivity edges for functional data and the 151 features for structural data. This approach was chosen for its sensitivity to heteroscedasticity, enabling the detection of group differences in variance. Multiple comparisons were corrected using the FDR method, with significant results identified at p < 0.05. We applied three additional corrections, either individually or in the following order: covariate regression, site correction, and sample balancing. To account for potential confounding effects, four covariates, including age, gender, parental income, and head motion, were regressed from each imaging feature in covariate-adjusted models. For site-corrected analyses, ComBat was performed using validated scripts (https://github.com/Jfortin1/ComBatHarmonization).^61^ In brief, ComBat is a popular tool for correcting for batch effects that aims to remove site-related variance from MRI data.^62–64^ For balanced analyses, we sought to investigate whether the increased variance observed in data from Black participants was due to the smaller number of Black compared to White participants. Racial groups were balanced by randomly selecting white participants to match the number of Black participants. Given the randomness of this selection process, results were repeated for 100 random iterations and reported as the median. Furthermore, we conducted a *χ*^2^ test to determine whether the number of high-variance edges are significantly different by race.

### Behavioral Measures

Thirty-six behavioral measures from the ABCD dataset were included in this analysis to capture a broad range of neurodevelopmental and mental health outcomes (listed in Table 3 and **Table S3**). These measures come from a previous work investigating racialized differences in neuroimaging predictive models.^41^ Beyond this, these measures were chosen for their relevance to brain-behavior associations, their utility in predicting neurodevelopmental trajectories, and their ability to capture a range of cognitive, emotional, and behavioral domains relevant to neurodevelopment and mental health outcomes.

### Racial Differences in Variance for Behavioral Measures

Behavioral measures were also assessed using Levene’s test to determine racial differences in variance. The same steps were used for the imaging data, excluding site correction, as the behavioral measures were standardized across sites.

### Cross-Validation Scheme

Predictive analyses were primarily performed using CPM with five-fold cross-validation. Folds were divided by family to avoid data leakage, ensuring that members of the same family were either in the training or test split. Prediction significance was assessed using permutation testing by shuffling the behavioral variable, repeating the cross-validation pipeline, and forming a null distribution across 500 permutations. The permutation p-value was defined as the proportion of permuted models achieving performance greater than or equal to the observed correlation between predicted and observed values.

Three distinct five-fold cross-validation pipelines were analyzed. First, we performed predictive modeling without any corrections. Second, we corrected for imbalanced data by randomly sampling participants such that an equal number of individuals were present by race. Third, we applied the imbalance correction as well as site correction via ComBat and regression of four covariates (age, self-reported sex, family income, and head motion).

### Assessment of Differences in Prediction Variance

For five-fold cross-validation results with at least (*r* > 0.1) and significant (p < 0.05) predictions, we considered the variance difference between predictions of Black and White participants. For each phenotype, we quantified racial differences in predictive dispersion using *var_diff = Var(ŷ_Black) − Var(ŷ_White)* where ŷ denotes pooled out-of-sample predictions across 5-fold cross-validation splits. Positive values indicate greater predictive dispersion among Black participants. Significance of each variance difference was determined by permutation testing, running 1000 permutations to shuffle race and recompute variance differences. Race labels were randomly permuted across participants while holding predicted values fixed, and *var_diff* was recomputed to generate a null distribution. Two-sided permutation p-values were calculated as the proportion of permuted |*var_diff*| values at least as extreme as the observed value. Furthermore, beyond significance of each individual variance difference, we considered whether the 20 models with modest and significant prediction performance generally had positive or negative variance differences. To do this, we performed a binomial test to calculate the significance of 16 values with positive *var_diff* among the 20 tested values in R (“binom.test(16, 20, p = 0.5, alternative = c(“two.sided”), conf.level = 0.95)”).

### Differences in Prediction Variance for Additional Models

We implemented the 5-fold cross-validation pipelines for three additional models: Kernel Ridge Regressor (KRR), Random Forest Regressor (RFR) and Artificial Neural Network (ANN). KRR combines ridge regression with the kernel trick to model nonlinear relationships.^65,66^ In this study, we used a radial basis function (RBF) kernel, with regularization parameter *α* controlling model complexity and kernel coefficient *γ*, governing the influence of individual training samples. These hyperparameters were selected empirically to balance bias and variance. RFR is an ensemble learning method that constructs multiple decision trees during training and outputs the average prediction of the individual trees.^67^ We configured the model with a fixed number of estimators, limited tree depth, and square-root feature sampling at each split to reduce overfitting and improve generalization. Both KRR and RFR implementations were obtained from the Scikit-learn library, which provides efficient and widely adopted implementations of classical machine learning algorithms.^68,69^ The ANN comprised three dense layers (64, 32, and 1 neurons) with dropout regularization (dropout rate = 0.2) applied after the first and second dense layers. ReLU activation functions were used in each hidden layer while linear activations were used in the output layer (**Table S7**).

### Tests of Heteroscedasticity for Brain-behavior Summary Scores

We conducted Levene’s test and the Breusch-Pagan test on brain-behavior summary scores to assess for variance differences by race in the brain-behavior relationship. Brain-behavior summary scores were formed using the 36 behavioral measures and rsfMRI connectivity. To form the scores, we followed the internal validation step of CPM.^70^ In brief, each imaging feature is correlated with the behavior of interest across all participants. Subsequently, the top 10% of features with the largest effect sizes are selected and summed together in individual participants to form a summary score for each participant. The summary scores were generated in the entire dataset (i.e., not cross-validated) because Levene’s test and the Breusch-Pagan test do not support cross-validation.

We then performed Levene’s test for the equality of variances, using absolute deviations of the data from their group means.^27,60,71^ We further assessed heteroskedasticity using the Breusch-Pagan test.^26^ For each behavioral phenotype, a linear regression model was fit with the CPM-derived total score as the independent variable and the observed behavioral measure as the dependent variable. Model residuals were extracted and tested for non-constant variance using the Breusch-Pagan Lagrange Multiplier procedure, as implemented in statsmodels library.^72^ A statistically significant result indicates violation of the homoskedasticity assumption and suggests that prediction error variance is not constant across observations.

### Simulation

To demonstrate the effect of heteroscedasticity on CPM performance, linear regression was performed on simulated data pooled between two groups of 1000 participants each. X data for group 1 was always set to have mean equal to zero and variance equal to 1, that is, μ x_1_ = 0 and σ ^2^x_1_ = 1. Y data for group 1 was always set to have mean equal to 0 and variance equal to 3, that is, μ Y_1_ = 0 and σ ^2^_Y1_ = 1. X and Y data for group two similarly always had zero mean (μ x_2_ = 0, μ Y_2_ = 0). However, σ ^2^x_2_ was allowed to take values 1, 1.1, 1.5, and 2, and σ ^2^_Y2_ was allowed to take values 3, 3.3, 4.5, and 6. For each pair of possible variance values for X_2_ and Y_2_, 100 iterations were performed. Unique data for both groups was generated in each iteration. To generate the X data for group 1, a vector was created with evenly spaced values between 0 and 9.99. Then, zero-mean Gaussian random noise with variance of 1 was added to each point, and the data was z-scored. To generate the X data for group 2, the same process was performed except that after z-scoring, the resulting data was multiplied by the square root of the desired variance. For both groups, Y was constructed as *Y*_*i*_ =*βX*_*i*_ +*ϵ*_*i*_, where *ϵ*_*i*_ is zero-mean Gaussian noise, and *β* was set to 0.5. The variance of the noise was set such that the variance of *Y*_*i*_ reached the desired level. Linear regression was then performed on the pooled data with an intercept. Predicted values, Z_1_ and Z_2_, were then calculated for each group. The mean squared error for each group was then calculated as the average squared difference between Y and Z within each group. Within each group, correlations between Y and Z were also calculated.

### Software

All analyses were conducted using Python and MATLAB for data preprocessing, statistical analysis, and CPM. Python packages such as scikit-learn,^73^ NumPy and SciPy were used for prediction and statistical tests, and pandas was employed for data management.^74^ Code is available on GitHub: https://github.com/mattrosenblatt7/neuroimaging_racial_heteroscedasticity/tree/main.

## Supplementary Material

Supplementary Files

This is a list of supplementary files associated with this preprint. Click to download.

- NNA95881VariancePredictionSupplementaryInformationv1.docx
- NNA95881SupplementaryTables.xlsx

## Figures and Tables

**Table 1:** Number of significant results in Levene’s test for imaging data. Based on the number of imaging features that had significantly higher variance for White and Black participants (including p < 0.05 and an FDR-corrected threshold), we conducted a *χ*^2^ test to determine whether the number of high-variance edges are significantly different by race. Ratios represent the count of features with greater variance in Black versus White participants; higher ratios indicate a stronger skew toward Black-variance dominance within that modality and correction type.

		*p* < 0.05					*All corrections (covariate regression, site correction, balanced sample sizes, FDR correction)*			
Modality	Total # of features	Total # of significantly variant features (% total features)	# higher variance (% total features, % significant features)		Ratio	*χ*^*2*^ (*p*)	Total # of significantly variant features (% total features)	# higher variance (% total features, % significant features)		*χ*^*2*^ (*p*)
			Black	White				Black	White	
**rsfMRI**	35778	5727 (16.01%)	3874 (9.98%, 62.37%)	2155 (6.02%, 37.63%)	1.65	350.60 (p < 1e-16)	2 (0.006%)	2 (0.006%, 100%)	0 (0%)	2 (p = 0.16)
**MID**	35778	8112 (22.67%)	7438 (20.79%, 91.69%)	674 (1.88%, 8.31% of)	11.03	5640.00 (p < 1e-16)	10 (0.03%)	10 (0.03%, 100%)	0 (0%)	10 (p = 1.56e-3)
**EN-Back**	35778	4451 (12.44%)	3076 (8.60%, 69.11%)	1375 (3.84%, 30.89%)	2.24	650.06 (p < 1e-16)	0 (0%)	0 (0%)	0 (0%)	NA
**SST**	35778	5807 (16.23%)	4911 (13.73%, 84.57%)	896 (2.50%, 15.43%)	5.48	2776.00 (p < 1e-16)	1 (0.003%)	0 (0.003%, 100%)	1 (0%)	1 (p = 0.32)
**DWI**	151	70 (46.36%)	15 (9.93%, 21.43% s)	55 (36.42%, 78.57%)	0.27	22.86 (p = 1.7e-6)	17 (11.26%)	17 (11.26%, 100%)	0 (0%)	17 (p = 3.74e-5)
**SMRI**	151	49 (32.45%)	25 (16.56%, 51.02%)	24 (15.89%, 48.98%)	1.04	.02 (p = 0.89)	0 (0%)	0 (0%)	0 (0%)	NA

**Table 2:** Number of significant results in Levene’s test for imaging data after corrections. We reported the number of imaging features that had significantly higher variance for White and Black participants (p < 0.05 threshold) for four conditions: 1) applying covariate regression to the imaging data, 2) applying covariate site correction to the imaging data, 3) balancing the imaging data to have an equal number of participants by race, and 4) applying all of the corrections 1–3. For any results with balancing (Cases 3–4), results were repeated for 100 random iterations and reported as the median. For the case with all corrections, we conducted a *χ*^2^ test to determine whether the number of high-variance edges are significantly different by race.

		Modality					
Corrections		rsfMRI	MID	EN-Back	SST	DWI	SMRI
Covariate regression	Total # of significantly variant edges (% total features)	5262 (14.71%)	7947 (22.21%)	3956 (11.06%)	5760 (16.1%)	17 (11.26%)	0 (0% of total features)
	# higher variance in Black participants (% total features, % of significantly different features)	3656 (10.22%, 69.48%)	7444 (20.81%, 93.67%)	2765 (7.73%, 69.89%)	5132 (14.34%, 89.1%)	11 (7.28%, 64.71%)	0 (0% of total features)
	# higher variance in White participants (% total features, % of significantly different features)	1606 (4.49%, 30.52%)	503 (1.41%, 6.33%)	1191 (3.33%, 30.11%)	628 (1.76%, 10.9%)	6 (3.97%, 35.29%)	0 (0% of total features)
Site correction	Total # of significantly variant edges (% total features)	3829 (10.7%)	5703 (15.94%)	3513 (9.82%)	4411 (12.33%)	87 (57.62%)	117 (77.48%)
	# higher variance in Black participants (% total features, % of significantly different features)	2218 (6.2%, 57.93%)	5067 (14.16%, 88.85%)	2365 (6.61%, 67.32%)	3689 (10.31%, 83.63%)	86 (56.95%, 98.85%)	117 (77.48%, 100.%)
	# higher variance in White participants (% total features, % of significantly different features)	1611 (4.5%, 42.07%)	636 (1.78%, 11.15%)	1148 (3.21%, 32.68%)	722 (2.02%, 16.37%)	1 (.66%, 1.15%)	0 (0%, 0%)
Balanced sample sizes	Total # of significantly variant edges (% total features)	4286 (11.98%)	5415 (15.13%)	3279 (9.16%)	3922.5 (10.96%)	49.5 (32.78%)	39 (25.83%)
	# higher variance in Black participants (% total features, % of significantly different features)	2667 (7.45%, 62.22%)	4844 (13.54%, 89.46%)	2155.5 (6.02%, 65.74%)	3128 (8.74%, 79.75%)	11.5 (7.61%, 23.23%)	18 (11.92%, 46.15%)
	# higher variance in White participants (% total features, % of significantly different features)	1619 (4.52%, 37.77%)	570.5 (1.59%, 10.54%)	1123.5 (3.14%, 34.26%)	794.5 (2.22%, 20.25%)	38 (25.16%, 76.77%)	21 (13.91%, 53.85%)
FDR correction	Total # of significantly variant edges (% total features)	875 (2.45%)	2409 (6.73%)	311 (0.87%)	678 (1.90%)	45 (29.80%)	29 (19.21%)
	# higher variance in Black participants (% total features, % of significantly different features)	614 (1.72%, 70.17%)	2305 (6.44%, 95.68%)	264 (0.74% 84.89%)	615 (1.72%, 90.71%)	12 (7.95%, 26.67%)	13 (8.61%, 44.83%)
	# higher variance in White participants (% total features, % of significantly different features)	261 (0.73%, 29.83%)	104 (0.29%, 4.32%)	47 (0.13%, 15.11%)	63 (0.18%, 9.29%)	33 (21.85%, 73.33%)	16 (10.60% 55.17%)
	Ratio	2.35	22.16	5.62	9.76	0.36	0.81

**Table 3:** Variance differences in behavioral measures between Black and White participants. The table presents results of Levene's test for variance differences across 36 behavioral measures, including the p-value (P), F-statistic (f), and standard deviations for Black (SD (Black)) and White (SD (White)) participants. The ratio of standard deviations (SD Ratio (Black / White)) quantifies the relative variability between the two groups, with values > 1 indicating greater variability among Black participants and values < 1 indicating greater variability among White participants. Measures with statistically significant variance differences (p < 0.05) are ordered by F-statistic. Sample sizes (N) vary across measures due to data availability. These results highlight racialized patterns of variability in cognitive, emotional, and behavioral domains, with most measures showing greater variability among Black participants.

Behavior	P	f	SD (Black)	SD (White)	SD Ratio (Black / White)	N
cbcl_scr_syn_rulebreak_r	6.05e-65	294.33	2.34	1.65	1.42	9,007
pgbi_p_ss_score	4.47e-59	266.57	3.52	2.44	1.44	9,005
nihtbx_flanker_uncorrected	3.00e-54	243.83	10.81	8.20	1.32	8,899
pps_y_ss_severity_score	2.79e-35	154.96	12.19	9.61	1.27	9,007
nihtbx_cardsort_uncorrected	1.68e-33	146.69	11.04	8.65	1.28	8,900
lmt_scr_rt_correct	2.04e-26	113.85	527.68	449.28	1.17	8,765
pps_y_ss_number	3.15e-22	94.50	3.91	3.36	1.16	9,006
upps_y_ss_positive_urgency	1.64e-19	82.00	3.20	2.83	1.13	9,001
bis_y_ss_bas_drive	4.80e-19	79.87	3.29	2.93	1.12	9,001
cbcl_scr_syn_aggressive_r	9.08e-19	78.60	5.01	4.16	1.21	9,007
lmt_scr_perc_correct	1.33e-17	73.27	0.15	0.17	0.91	8,768
cbcl_scr_syn_attention_r	1.26e-16	68.78	3.82	3.37	1.13	9,007
cbcl_scr_syn_social_r	9.51e-15	60.20	2.52	2.19	1.15	9,007
upps_y_ss_lack_of_planning	1.45e-13	54.81	2.57	2.32	1.11	9,001
nihtbx_fluidcomp_uncorrected	1.31e-12	50.46	11.14	9.82	1.13	8,845
nihtbx_list_uncorrected	1.74e-12	49.90	12.63	11.27	1.12	8,875
upps_y_ss_negative_urgency	4.37e-12	48.08	2.85	2.58	1.11	9,001
cbcl_scr_syn_withdep_r	4.56e-10	38.94	1.89	1.65	1.15	9,007
nihtbx_totalcomp_uncorrected	1.04e-09	37.33	9.23	8.22	1.12	8,845
pea_wiscv_trs	4.38e-08	30.02	3.93	3.60	1.09	8,838
bis_y_ss_bis_sum	4.76e-08	29.86	4.00	3.65	1.10	9,001
pea_ravlt_sd_trial_vi_tc	1.45e-07	27.70	3.16	2.92	1.08	8,862
nihtbx_reading_uncorrected	3.09e-07	26.23	7.18	6.45	1.11	8,895
cbcl_scr_syn_thought_r	3.10e-07	26.23	2.40	2.14	1.12	9,007
upps_y_ss_sensation_seeking	5.65e-05	16.23	2.82	2.64	1.07	9,001
nihtbx_pattern_uncorrected	1.32e-04	14.63	15.07	14.21	1.06	8,889
nihtbx_picture_uncorrected	2.71e-04	13.27	11.43	12.01	0.95	8,897
bis_y_ss_bas_fs	4.43e-04	12.35	2.79	2.58	1.08	9,001
nihtbx_cryst_uncorrected	2.84e-03	8.91	6.84	6.52	1.05	8,883
cbcl_scr_syn_anxdep_r	3.99e-03	8.29	3.02	3.10	0.97	9,007
cbcl_scr_syn_somatic_r	6.89e-03	7.31	2.08	1.92	1.08	9,007
lmt_scr_efficiency	0.02	5.14	0.07	0.07	0.97	8,765
upps_y_ss_lack_of_perseverance	0.04	4.41	2.33	2.22	1.05	9,001
pea_ravlt_ld_trial_vii_tc	0.27	1.23	3.20	3.09	1.04	8,824
bis_y_ss_bas_rr	0.27	1.19	2.97	2.88	1.03	9,001
nihtbx_picvocab_uncorrected	0.60	0.28	7.60	7.57	1.00	8,906

**Table 4:** Variances differences in predicted values by race. The table presents results for predictive models using rsfMRI and 36 behavioral measures. The correlation between predicted and observed values (r) was tested for significance with permutation testing (P). For values with at least modest (*r* > 0.1) and significant (p < 0.05) predictions, variance differences by racial group were assessed (ΔVariance), reflecting the variance of predicted values in Black participants minus the variance in White participants. Significance of variance differences was assessed by randomly shuffling the race variable for 1000 permutations while holding the predicted value constant.

Behavior	r	P	ΔVariance	P_ΔVariance_
nihtbx_cryst_uncorrected	0.262	0.001	0.016	0.011
nihtbx_totalcomp_uncorrected	0.253	0.001	0.009	0.083
nihtbx_picvocab_uncorrected	0.248	0.001	0.015	0.008
nihtbx_reading_uncorrected	0.204	0.001	0.005	0.174
nihtbx_fluidcomp_uncorrected	0.192	0.001	0.003	0.237
pea_wiscv_trs	0.179	0.001	0.001	0.432
nihtbx_list_uncorrected	0.164	0.001	0.000	0.507
nihtbx_cardsort_uncorrected	0.152	0.001	0.002	0.29
lmt_scr_perc_correct	0.153	0.001	−0.002	0.763
pea_ravlt_ld_trial_vii_tc	0.141	0.001	0.002	0.272
pps_y_ss_number	0.137	0.001	−0.004	0.936
pea_ravlt_sd_trial_vi_tc	0.137	0.001	0.001	0.303
pps_y_ss_severity_score	0.133	0.001	−0.003	0.843
bis_y_ss_bas_drive	0.125	0.001	0.005	0.079
nihtbx_picture_uncorrected	0.124	0.001	0.000	0.473
upps_y_ss_positive_urgency	0.123	0.001	0.001	0.345
cbcl_scr_syn_attention_r	0.118	0.001	−0.004	0.963
cbcl_scr_syn_rulebreak_r	0.116	0.001	0.019	0.001
nihtbx_pattern_uncorrected	0.114	0.001	0.004	0.134
pgbi_p_ss_score	0.109	0.001	0.013	0.003
bis_y_ss_bas_rr	0.0981	0.001	-	-
nihtbx_flanker_uncorrected	0.096	0.001	-	-
lmt_scr_efficiency	0.094	0.001	-	-
upps_y_ss_sensation_seeking	0.084	0.001	-	-
cbcl_scr_syn_thought_r	0.082	0.003	-	-
cbcl_scr_syn_social_r	0.081	0.001	-	-
cbcl_scr_syn_aggressive_r	0.071	0.001	-	-
lmt_scr_rt_correct	0.066	0.005	-	-
cbcl_scr_syn_anxdep_r	0.061	0.001	-	-
bis_y_ss_bas_fs	0.060	0.005	-	-
upps_y_ss_lack_of_perseverance	0.051	0.007	-	-
upps_y_ss_negative_urgency	0.050	0.011	-	-
upps_y_ss_lack_of_planning	0.050	0.021	-	-
cbcl_scr_syn_withdep_r	0.035	0.097	-	-
cbcl_scr_syn_somatic_r	0.030	0.149	-	-
bis_y_ss_bis_sum	0.007	0.769	-	-

## Data Availability

The data supporting the findings of this study were obtained from the Adolescent Brain Cognitive Development (ABCD) Study. Due to restrictions, the data are available upon reasonable request from the corresponding author or from the ABCD Study Data Access portal. The source data used in this study were openly available prior to its initiation. The ABCD data used in this report came from the fast track data release 5.1, which is accessible to qualified researchers through the NIMH Data Archive (NDA). The raw data are available at https://nda.nih.gov/study.html?id=2313, and the data dictionary for ABCD can be found at https://datadict.abcdstudy.org/. Additional details about the measures assessed for the ABCD study are provided at https://wiki.abcdstudy.org/release-notes/start-page.html. Instructions for obtaining NDA data use certification are available at https://nda.nih.gov/nda/access-data-info.
